# Views of Menopause and Hormone Therapy Associations with Hormone Therapy Use in US Women Aged 50–79

**DOI:** 10.1177/26884844251372014

**Published:** 2025-09-02

**Authors:** Sushmita Chopra, Dokyung Yoon, Teal Eich

**Affiliations:** The Davis School of Gerontology, University of Southern California, Los Angeles, California, USA.

**Keywords:** menopause, hormone therapy, aging, perception

## Abstract

**Objective:**

To explore symptoms, knowledge levels, perceptions, and use related to menopause and hormone therapy (HT) and to examine the factors associated with HT use and HT perceptions in perimenopausal and postmenopausal women.

**Materials and Methods:**

We used a sample of 98 perimenopausal and postmenopausal women who aged 50–79 and participated in the Sex, ApoE-4, γ-aminobutyric acid, and Episodic memory (SAGE) study (*M*_age_ = 64.24, *standard deviation* = 7.49). We applied a series of bivariate Firth logistic regressions to examine the associations of each variable of interest with hormone therapy (HT) use and perceptions.

**Results:**

Overall, 89.90% reported positive perceptions of menopause, and 85.71% had positive perceptions of HT. One-third (32.65%) of the sample had used HT. Hot flashes (72.4%) were the most reported menopausal symptom. Logistic regression analyses showed that age, race/ethnicity, current drinking status, menopausal knowledge levels, and vasomotor and genitourinary symptoms were significantly associated with HT use, while race/ethnicity, current drinking status, menopausal knowledge levels, and genitourinary symptoms were also linked to positive HT perceptions. Hispanic participants reported lower menopausal knowledge, less positive HT perceptions, and lower HT use.

**Conclusions:**

The majority of women in the SAGE cohort reported positive perceptions of both menopause and HT. Race/ethnicity, along with current drinking status, menopausal knowledge levels, and genitourinary symptoms, were consistently associated with both HT use and HT perceptions. Ethnic differences in menopausal knowledge levels, HT perceptions, and HT use are also discussed.

## Introduction

Menopause is a major reproductive event experienced by every woman who lives long enough. The World Health Organization defines menopause as the loss of ovarian follicular function, evidenced by amenorrhea for a consecutive period of 12 months, whether induced or naturally occurring. Menopause, which on average occurs around age 51 in American women, is preceded by a relatively long period of changes in endocrine hormones including 17β estradiol (E2), progesterone (P4), testosterone, luteinizing hormone, follicle-stimulating hormone (FSH), sex hormone-binding globulin (SHBG; a protein), anti-Müllerian hormone (AMH), and inhibin B. Longitudinal data from the Study of Women’s health Across the Nation (SWAN)^1^ show that during the perimenopause, or the transitional state leading up to the final menstrual period, E2 and P4 fluctuate dramatically, with steep declines occurring after menopause. Before these changes (and approximately 6 to 10 years before the final menses), FSH levels begin to rise in response to declines in inhibin B levels, which in turn impacts SHBG levels, changing the balance of free versus bound circulating hormones. FSH levels continue to rise sharply until 2 years following the onset of menopause, after which time these levels stabilize. For this reason, FSH, AMH, and inhibin B, and not E2 or P4, are considered supportive criteria in reproductive staging according to the gold standard Stages of Reproductive Aging Workshop (STRAW) + 10 criteria.^2^

Data from SWAN indicate that between 50% and 82% of women experience vasomotor symptoms associated with menopause, including hot flashes and night sweats.^3^ Other commonly experienced symptoms include mood and sleep disturbances and vaginal dryness. In addition to impacting quality of life,^4^ vasomotor symptoms are associated with an increased risk of cardiovascular disease.^5^ Hormone therapy (HT) was developed in the 1930s and 1940s to alleviate symptoms associated with menopause.^6^ In the 1960s and 1970s, unopposed conjugated equine estrogen (CEE) therapy (FDA approved in 1942 and sold under the trade name Premarin) was the most prescribed drug for American women.^7^ The publication of interim results of the clinical trial arm of the Women’s Health Initiative (WHI)^8^ which reported higher rates of breast cancer, stroke, pulmonary embolism, and myocardial infarction^,^ in postmenopausal women who received combination therapy (Prempro, a combination CEE and medroxyprogesterone) as compared to placebo, resulted in a black box warning. Following this, HT prescriptions declined by almost 80%,^9^ current users of HT declined by 28% while new users of the therapy declined by 50%^10^ and overall HT use decreased from ∼43% to 11% in women aged 45–74.^11^

More recently, a number of studies^12–17^ have supported the “timing hypothesis,” which posits that risks associated with HT depend on when it is initiated relative to the onset of menopause. Results from the Kronos Early Estrogen Replacement Study, a prospective, randomized, double-blind trial of the effect of CEE and transdermal 17β-estradiol [t-E2]) as compared to placebo on atherosclerosis progression as measured by changes in carotid artery intima-media thickness (CIMT) as well as menopausal symptoms in recently postmenopausal women (aged 42–58 and within 6 months to 3 years of menopause) found that HT was not associated with an increased rate of CIMT progression, and instead trended, in the o-CEE users, to be protective. HT was also highly effective at reducing symptoms associated with hot flushes, improving sleep, and preserving bone health in this study.^18^ Results from the Danish Osteoporosis Prevention Study, which followed women randomized within 2–24 months of menopause to triphasic estradiol and norethisterone acetate (if they had a uterus) or estradiol (if they did not) versus placebo found that women receiving HT had a reduced risk of mortality, heart failure, and myocardial infarction and did not show an increased risk of cancer, venous thromboembolism or stroke.^16^ Data from the Early versus Late Intervention Trial with Estradiol found that women treated with HT within 6 years of menopause, as compared to those treated 10+ years after, had reduced CIMT.^14^ For women in this trial treated with HT 10+ years following menopause, CIMT rates were equivalent to placebo. Two related studies showed no increases in atherosclerosis progression in either postmenopausal women without coronary heart disease treated with unopposed 17β-estradiol (in the Estrogen in Prevention of Atherosclerosis Trial [EPAT])^19^ and postmenopausal women with established coronary heart disease treated with either unopposed or combined HT (in the Women’s Estrogen Lipid-Lowering Hormone Atherosclerosis Regression Trial).^20^

These data, along with others,^21–24^ underscore an evolution in the understanding of the safety and efficacy of HT, highlighting the particular importance of early initiation for achieving safety and even benefits, especially for cardiovascular health and menopause symptom management. Given this, in the current study, we were interested in factors that influence the decision to use HT, and in particular, whether perceptions of menopause and HT are associated with differential patterns of HT use.

Given the potential impact of HT on women’s health outcomes during and after menopause, it is essential to understand how women perceive both menopause and HT, as well as how they access and evaluate related information—particularly in light of shifts in public perception and clinical guidance following the WHI findings. A number of studies have queried women about their views on menopause. A large (*n* = 3150) online study of women aged 40 and older found that ⅓ of respondents were accepting, while a third reported “dreading it.”^25^ In this study, more than half (60%) of the respondents said they did not feel informed at all about menopause. Sources of information endorsed included friends and the internet in 68.2% of women. In a UK-based online study of 829 postmenopausal women, found that 49% felt completely uninformed about menopause before experiencing it.^26^ According to this study, >60% of women researched menopause only after onset, with 49.8% consulting friends and 33.1% relying on social media platforms for information. Prior to menopause, 18% of women were accepting of it, 15.8% dreaded it, and only 5.1% looked forward to it. In the postmenopausal group, 38.1% found the experience difficult, 24.6% very difficult, while 20.7% found it manageable. Another recent study that surveyed women across the menopausal transition about their views of menopause reported that women at all ages felt that information regarding menopause should be taught in school (but wasn’t), and that they lacked knowledge about menopause.^27^ Furthermore, the younger (pre or perimenopausal) women expressed more negative attitudes toward menopause than did the postmenopausal women, replicating previous work,^28,29^ including a systematic review of the extant literature.^30^ Thus, women in general seem to have positive views of menopause, albeit these views may depend on reproductive stage, but they felt they lacked adequate knowledge about it. These studies, however, which are mostly from after the WHI, did not query HT use, and thus, the impact of perceptions of menopause on use was not investigated.

Views on HT use have also been investigated. One study published prior to the WHI study reported that the perception of being adequately informed about the benefits of HT by a medical provider was associated with a higher likelihood of having a positive attitude toward HT.^31^ Griffiths reported that 53% of their 1,649 respondents (ranging in age from 20 to 69) agreed the media portrayed HT as positive, but 30% felt that it was unhelpful and 17% thought that it was incorrect.^32^ On the contrary, women reported that information from general practitioners and practice nurses was the most important in helping women decide about initiating HT, suggesting a critical role of medical professions in both providing knowledge and influencing decisions. Another study found that 89.3% of respondents had a positive view of HT due to its impact on menopausal symptomatology.^33^ However, this study also reported that women were more likely to get information about the risks and benefits of HT from mass media or a personal contact (48%) than from a health professional (41%). Thus, even before the WHI study, sources of information about HT were seen as influential in shaping women’s decisions to start therapy, but women still felt they lacked knowledge.

A number of studies have also been conducted since the original WHI study. Salame et al. found that while more than half (57.9%) of 40+ year old women who responded to a survey supported the use of HT, 47.9% did not know when to use it. In their sample, 51.6% of participants had been prescribed HT, and the belief that HT treats menopausal symptoms predicted a positive perception of HT.^34^ This study did not ask about sources of knowledge, however. A cross-sectional study by McIntosh and Blalock, including 97 menopausal women who had previously used HT reported that 100% of the participants had heard about the WHI data.^35^ Slightly more than half (52%) indicated that the WHI results had directly impacted their usage of HT, and in those who did, trust in physicians significantly decreased. Schaller and Malhotra reported that attitudes toward HT significantly predicted the decision to start or continue HT, with both feelings and beliefs about HT showing independent influences on HT usage.^36^ Beliefs about what your social circle thinks was the second strongest predictor of the decision to use HT in this study.

Although prior studies have assessed women’s general attitudes toward menopause and HT, few have directly examined how perceptions of menopause may be linked to decisions about HT use. Moreover, while several investigations have highlighted women’s lack of knowledge about menopause and HT, and pointed to nonmedical sources—such as peers, media, and the internet—as sources of information, there remains a critical gap in our understanding of how these factors interact in shaping actual patterns of HT uptake. The timing hypothesis of HT initiation supports a more nuanced view of the risks and benefits of HT, and thus clarifying how women’s beliefs and informational environments influence their choices as a function of their age is essential. In the current study, thus, we report the results of self-reported questionnaire data from 50 to 79 year old women’s perceptions of menopause and HT, sources of information, as well as HT use in order to explore the associations among menopausal perceptions, attitudes toward HT, sources of information, and HT use in a diverse sample of peri- and postmenopausal women.

## Methods

### Participants

Here, we present preliminary results from a sample of 128 total women who were recruited to the Sex, ApoE-4, γ-aminobutyric acid (GABA), and Episodic memory (SAGE) study as of January 31, 2025. The SAGE study investigates the effects of sex on age-related changes in episodic memory, hippocampal activity, and hippocampal GABA concentration. To be eligible to participate in the SAGE study, participants had to have a minimum of 9 years of formal education, be fluent in spoken and written English, weigh at least 110 lbs, and be willing and eligible to complete an MRI scan and a blood draw. Participants were not eligible if they had MRI contraindications, were using medications that impact the GABAergic system, were currently nursing/lactating or pregnant, had been pregnant in the last year, or had a diagnosed neurological disorder.

Community-dwelling participants were recruited to the SAGE study from two sources: the University of Southern California School of Gerontology’s Healthy Minds participant pool and the Banner Alzheimer’s Institute’s Genematch program (NCT02564692; more details can be found in Langbaum et al.).^37^ All participants in the SAGE study complete a battery of clinical, health, psychosocial, and environmental questionnaires, which include the variables used in the current report (described below), neuropsychological testing, structural, functional, and spectroscopic brain imaging, and biospecimen collection, including plasma, serum, and urine.

### Variables

The menopause-related variables were derived from responses to the Menopause Health Questionnaire developed by the North American Menopause Society, which includes questions related to gynecological history (section 6), medication history (section 10), menopausal symptoms (section 13), and perceptions of menopause and HT (section 15).

In the current study, menstrual status was self-reported using the question, “How would you describe your current menstrual status?” Participants were asked to select premenopausal (defined as “before menopause, having regular periods”), perimenopausal (“changes in period, but have not gone 12 months in a row without a period”), or postmenopausal (“after menopause, no periods in the last 12 months”). Perceptions regarding menopause were coded as a binary variable (0: negative, 1: positive), and other responses were treated as missing. Subjective knowledge of menopause was assessed with the question, “How would you rate your knowledge of menopause?” Responses ranged from “little” to “very good” but were later coded as binary (0: little or fair, 1: moderately good or very good). Regarding sources of information about menopause, each option (books, internet, magazines, friends, TV, and healthcare providers) was assessed separately with binary responses (0: no, 1: yes). Thirty-three symptoms related to menopause were asked whether each symptom currently or previously bothered respondents using applicable statements (e.g., “I have hot flashes,” “I have night sweats,” and “I have difficulty getting to sleep”). Responses ranged from “not at all” to “extremely.” The total number of menopausal symptoms was calculated by assigning a value of 0 if the response was “not at all” and 1 for all other responses. Menopausal symptoms were categorized into (1) vasomotor, (2) sleep, (3) somatic, (4) mood, (5) cognitive, (6) genitourinary, and (7) libido. Each category was assigned a binary value (0: if none of the symptoms in the category bothered respondents, 1: if at least one symptom in the category bothered them). HT use was assessed with the binary question, “Have you taken HT?” (0: no, 1: yes). Views regarding HT were assessed by the question “What are your current views regarding HT for menopause?” and were coded as either 0 (“Negative. I don’t support the use of HT”) or 1 (“Positive. HT is appropriate for some women”). Other menopause-related variables include age at menarche (years) and age at the last period (years).

Other covariates included self-reported age (years), race/ethnicity (1: non-Hispanic White, 2: non-Hispanic Black, 3: Hispanic, 4: non-Hispanic Others [including American Indian, Alaskan Native, and Multiracial]), educational attainment (0: up to some college, 1: college degree or above), marital/partnership status (0: never married/divorced/widowed/separated, 1: married/living with partner), current working status (0: no, 1: yes), regular exercise status (0: never/rarely/occasionally, 1: at least three times a week/almost daily), current smoking status (0: no, 1: yes), current drinking status (0: no, 1: yes), and BMI class (1: underweight, 2: healthy weight, 3: overweight, 4: obesity) calculated using participants’ height and weight.

### Statistical analysis

For continuous variables, we report means and standard deviation, as they followed a normal distribution (confirmed by Shapiro–Wilk tests). For categorical variables, proportions are reported. For bivariate comparisons, a chi-square test (or Fisher’s exact test when cell counts were less than 5) and the Student’s *t-*test were used.

Due to the small sample size in our study, data separation issues occurred. To address these issues, we utilized Firth logistic regression, which applies a penalized likelihood-based method.^38^ A series of simple Firth logistic regressions were conducted for each variable with respect to the outcome variables (HT use and HT perceptions). The statistical analysis was performed using STATA 17.0, and Firth logistic regression was employed using “firthlogit” syntax. A two-sided *p* value of <0.05 was considered statistically significant.

## Results

Among 128 females in our study, those younger than 50 and older than 79 years were excluded (*n* = 6). Additionally, only those whose self-reported menstrual status as peri- or postmenopausal were included (*n* = 2 premenopausal women were excluded). Among peri- or postmenopausal females, we included only those whose menopause was spontaneous (“natural”) and excluded participants whose menopause was induced by surgery (surgical removal of both ovaries), chemo- or radiation therapy (*n* = 15). Lastly, we excluded those who were missing data on any variables of interest (*n* = 7), allowing for complete-case analysis with a final sample size of 98 females.

**FIG. 1.**
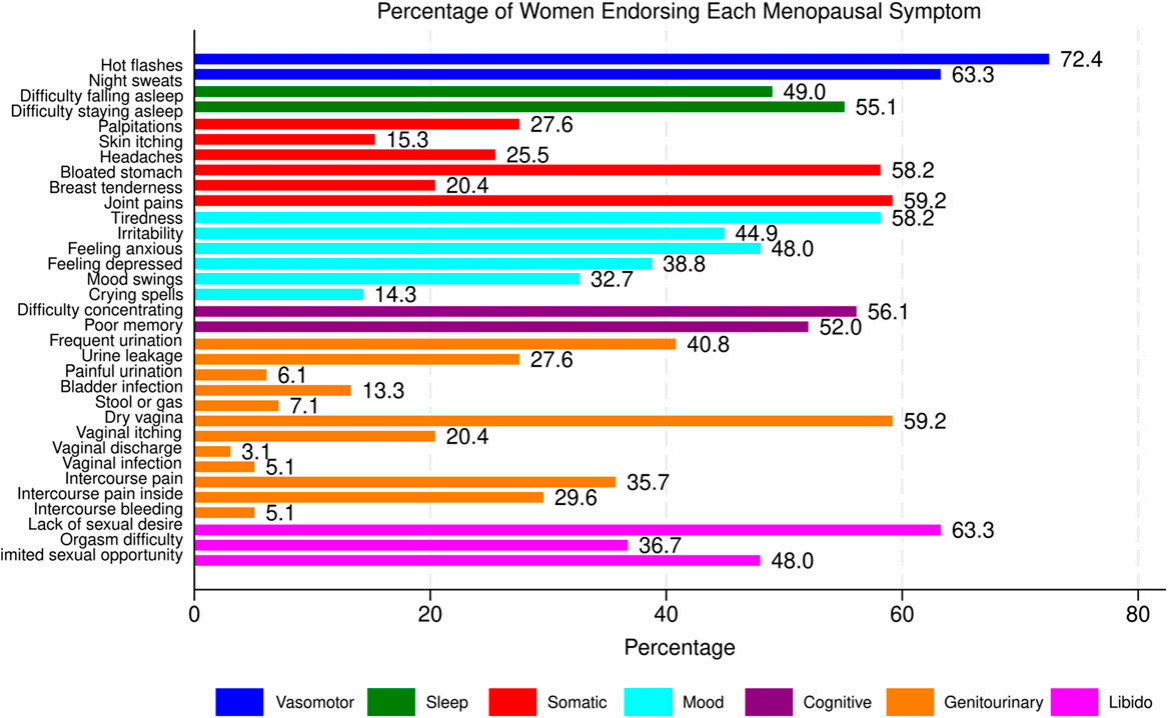
Menopausal symptoms experienced.

**FIG. 2.**
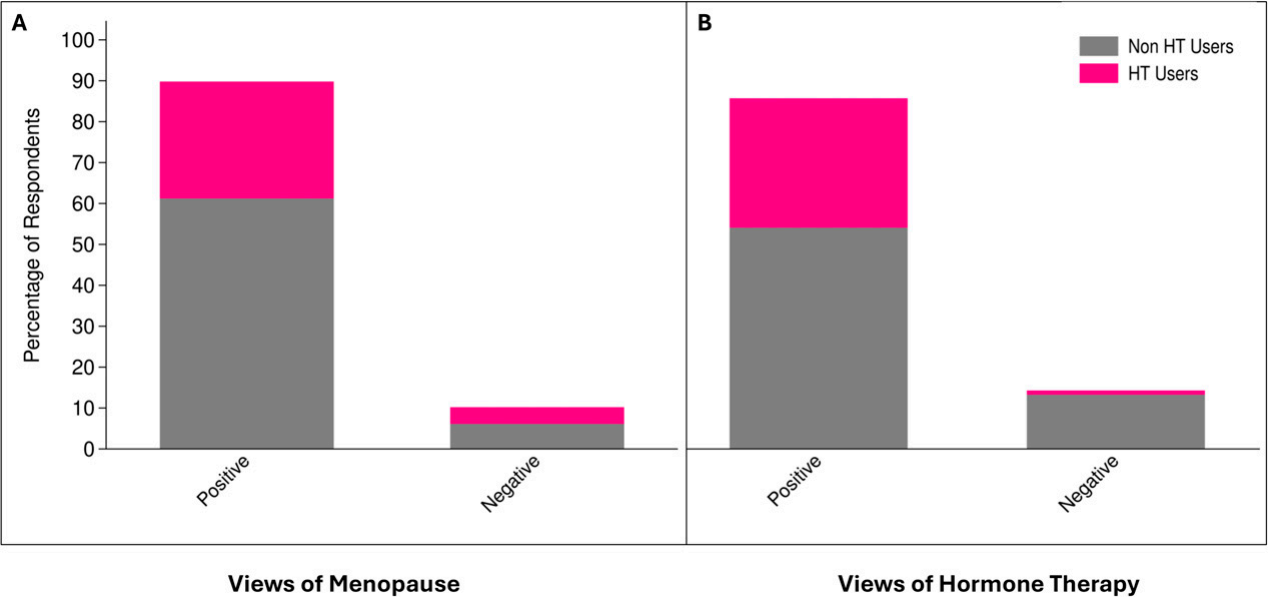
Views of **(A)** menopause and **(B)** hormone therapy, as a function of hormone therapy use.

As is shown in Table 1, the average age of the final sample was 64.24 years (Standard Deviation [*SD*] = 7.49). About two-thirds of the sample identified as non-Hispanic White (66.33%), followed by Hispanic, non-Hispanic Other, and non-Hispanic Black participants. The sample was highly educated, with a majority of the sample having attained a college degree or higher (71.43%). Half were married or living with a partner (50%). The mean age at menarche was 12.68 years (*SD* = 1.72), while the mean age at the last period among postmenopausal participants was 50.66 years (*SD* = 4.92). Most participants self-identified as postmenopausal (92.63%). Across the sample, a total of 2,939 menopausal symptoms were reported. As can be seen in Figure 1, hot flashes were the most commonly reported symptom, with 72.4% of females endorsing it. One-third (32.65%) of women reported having ever used HT for menopausal symptoms. Nearly, all women in our sample (89.80%) had positive perceptions regarding menopause, and 85.71% reported positive perceptions about HT. Among women with positive perceptions of menopause, 31.82% had used HT. Among those with negative perceptions regarding menopause, 40% had used HT (Fig. 2A). Among women who had positive perceptions of HT, 36.90% had used it. Among those with negative perceptions regarding HT use, 7.14% were ever users of HT (Fig. 2B).

**Table 1.** Descriptive Statistics

	All (*n* = 98)	HT use		*p*	HT perceptions		*p*
		Yes (*n* = 32)	No (*n* = 66)		Positive (*n* = 84)	Negative (*n* = 14)	
Age (years)	64.24 (7.49)	67.78 (7.38)	62.53 (6.96)	^***^	64.73 (7.47)	61.36 (7.17)	
Race/ethnicity				^***^			
NH White	65 (.66)	30 (.94)	35 (.53)		59 (.70)	6 (.43)	
NH Black	7 (.07)	1 (.03)	6 (.09)		6 (.07)	1 (.07)	
Hispanic	14 (.14)	1 (.03)	13 (.20)		9 (.11)	5 (.36)	
NH Others	12 (.12)	—	12 (.18)		10 (.12)	2 (.14)	
College degree or above	70 (.71)	26 (.81)	44 (.67)		62 (.74)	8 (.57)	
Married or living with partner	49 (.50)	19 (.59)	30 (.45)		41 (.49)	8 (.57)	
Currently working	28 (.29)	8 (.25)	20 (.30)		23 (.27)	5 (.36)	
Regularly exercising	79 (.81)	27 (.84)	52 (.79)		67 (.80)	12 (.86)	
Currently smoking	3 (.03)	—	3 (.05)		3 (.04)	—	
Currently drinking	64 (.65)	27 (.84)	37 (.56)	^**^	59 (.70)	5 (.36)	^*^
BMI Class							
Underweight	2 (.02)	—	2 (.03)		2 (.02)	—	
Healthy weight	51 (.52)	22 (.69)	29 (.44)		46 (.55)	5 (.36)	
Overweight	27 (.28)	6 (.19)	21 (.32)		19 (.23)	8 (.57)	
Obesity	18 (.18)	4 (.13)	14 (.21)		17 (.20)	1 (.07)	
Postmenopausal	91 (.93)	31 (.97)	60 (.91)		79 (.94)	12 (.86)	
Age at menarche (years)	12.68 (1.72)	12.94 (1.78)	12.56 (1.69)		12.67 (1.70)	12.79 (1.93)	
Age at last period (years)	50.66 (4.92)	50.86 (4.64)	50.55 (5.10)		50.72 (5.06)	50.18 (4.00)	
Subjective menopausal knowledge				^**^			^**^
Little knowledge	2 (.02)	—	2 (.03)		2 (.02)	—	
Fair	34 (.35)	12 (.38)	22 (.33)		25 (.30)	9 (.64)	
Moderately good	17 (.17)	—	17 (.26)		13 (.15)	4 (.29)	
Very good	45 (.46)	20 (.63)	25 (.38)		44 (.52)	1 (.07)	
Positive menopause perceptions	88 (.90)	28 (.88)	60 (.91)		76 (.90)	12 (.86)	
Ever use of HT	32 (.33)	—	—		31 (.37)	1 (.07)	^*^
Positive HT perceptions	84 (.86)	31 (.97)	53 (.80)	^*^	—	—	

^*^p < 0.05, ^**^p < 0.01, ^***^p < 0.001.

Mean (*standard deviation*) for age, age of menarche, and age at last period. *N* (percentage) for all other variables. Age at the last period was assessed only among postmenopausal females in the sample.

BMI, body mass index; HT, hormone therapy; NH, non-Hispanic.

Compared to non-HT users, HT users tended to be older, more likely to consume alcohol, and had more positive perceptions regarding HT use. Furthermore, compared to those with negative perceptions about HT, those with positive perceptions were more likely to consume alcohol and to have used HT. Subjective menopausal knowledge was highest for the response “very good” (45.92%). Among HT users, 87.5% had positive and 12.5% had negative perceptions toward menopause, while among nonusers, 90.91% had positive and 9.09% had negative perceptions. Furthermore, among HT users, 96.88% had positive and 3.13% had negative perceptions toward HT, while among nonusers, 80.30% had positive and 19.70% had negative perceptions.

A series of bivariate Firth logistic regressions was conducted separately for HT use (Table 2, left column) and HT perceptions (Table 2, right column). The results of the regression for HT use indicated that each 1-year increase in age was associated with 11% higher odds of using HT (OR = 1.11, 95% confidence interval [CI] = [1.04–1.18], *p* < 0.01). Compared to non-Hispanic Whites, Hispanics had 87% lower odds of using HT (OR = 0.13, 95% CI = [0.02–0.75], *p* < 0.05), and non-Hispanic Others had 95% lower odds of using HT (OR = 0.05, 95% CI = [0.00–0.82], *p* < 0.05). Current drinkers had 3.93 times higher odds of having ever used HT (OR = 3.93, 95% CI = [1.40–11.07], *p* < 0.01) compared to current nondrinkers. In addition, compared to those with “moderately good” levels of menopausal knowledge, both participants with “fair” (OR = 19.44, 95% CI = [1.08–351.59], *p* < 0.05) and “very good” (OR = 28.14, 95% CI = [1.59–496.56], *p* < 0.05) levels of menopausal knowledge showed higher odds of ever using HT. Furthermore, participants with vasomotor symptoms (OR = 19.23, 95% CI = [1.11–334.12], *p* < 0.05) and genitourinary symptoms (OR = 5.85, 95% CI = [1.02–33.49], *p* < 0.05) demonstrated higher odds of ever using HT compared to those without these symptoms.

**Table 2.** Results of Simple Firth Logistic Regressions

	HT use	HT perceptions
	OR [95% CI]	OR [95% CI]
Age (years)	1.11** [1.04–1.18]	1.06 [0.98–1.15]
Race/ethnicity (ref: NH White)		
NH Black	0.27 [0.04–1.69]	0.47 [0.07–3.33]
Hispanic	0.13* [0.02–0.75]	0.19* [0.05–0.71]
NH Others	0.05* [0.00–0.82]	0.46 [0.09–2.27]
College degree or above (ref: up to some college)	2.06 [0.76–5.58]	2.12 [0.69–6.57]
Married or living with partner (ref: never married/divorced/widowed/separated)	1.73 [0.74–4.02]	0.73 [0.24–2.21]
Currently working (yes) (ref: no)	0.79 [0.31–2.01]	0.66 [0.21–2.09]
Regularly exercising (yes) (ref: no)	1.38 [0.47–4.08]	0.77 [0.18–3.31]
Currently smoking (yes) (ref: no)	0.28 [0.01–5.57]	1.25 [0.06–25.40]
Currently drinking (yes) (ref: no)	3.93** [1.40–11.07]	4.03* [1.28–12.70]
BMI class (ref: underweight)		
Healthy weight	3.81 [0.17–83.44]	1.69 [0.07–39.95]
Overweight	1.51 [0.06–35.65]	0.46 [0.02–10.62]
Obesity	1.55 [0.06–38.65]	2.33 [0.07–74.55]
Age at menarche (years)	1.13 [0.89–1.45]	0.96 [0.69–1.33]
Subjective menopausal knowledge (ref: moderately good)		
Little	7.00 [0.11–438.68]	1.67 [0.07–41.64]
Fair	19.44* [1.08–351.59]	0.89 [0.24–3.29]
Very good	28.14* [1.59–496.56]	9.89* [1.41–69.20]
Menopausal symptoms		
Vasomotor symptoms (ref: no)	19.23* [1.11–334.12]	3.55 [0.96–13.22]
Sleep symptoms (ref: no)	2.44 [0.93–6.37]	1.95 [0.60–6.33]
Somatic symptoms (ref: no)	0.46 [0.15–1.40]	2.04 [0.52–7.97]
Mood symptoms (ref: no)	1.34 [0.45–3.99]	2.24 [0.64–7.86]
Cognitive symptoms (ref: no)	1.06 [0.43–2.61]	0.97 [0.29–3.28]
Genitourinary symptoms (ref: no)	5.85^*^ [1.02–33.49]	5.60^**^ [1.54–20.36]
Libido symptoms (ref: no)	1.60 [0.54–4.70]	1.91 [0.55–6.63]
Positive menopause perceptions (ref: negative)	0.68 [0.19–2.45]	1.80 [0.39–8.33]
Positive HT perceptions (ref: negative)	5.30 [0.93–30.29]	—

^*^p < 0.05, ^**^p < 0.01.

CI, confidence interval.

For HT perceptions, the regression results indicated that Hispanics had 81% lower odds of viewing HT use as positive compared to non-Hispanic Whites (95% CI = [0.05–0.71], *p* < 0.05). On the contrary, current drinkers had 4.03 times higher odds of viewing HT use as positive compared to current nondrinkers (95% CI = [1.28–12.70], *p* < 0.05). In addition, compared to those with “moderately good” levels of menopausal knowledge, participants with “very good” level of menopausal knowledge had 9.89 times higher odds of having a positive perception of HT (95% CI = [1.41–69.20], *p* < 0.05). Also, compared to those with “very good” levels of menopausal knowledge, participants with “fair” (OR = 0.09, 9 5% CI = [0.02–0.54], *p* < 0.01) and “moderately good” (OR = 0.10, 95% CI = [0.01–0.71], *p* < 0.05) levels of menopausal knowledge were less likely to have positive perceptions toward HT. Furthermore, participants with genitourinary symptoms trended toward having more positive perceptions toward HT compared to those without these symptoms (OR = 5.60, 95% CI = [1.54–20.36], *p* < 0.10).

**FIG. 3.**
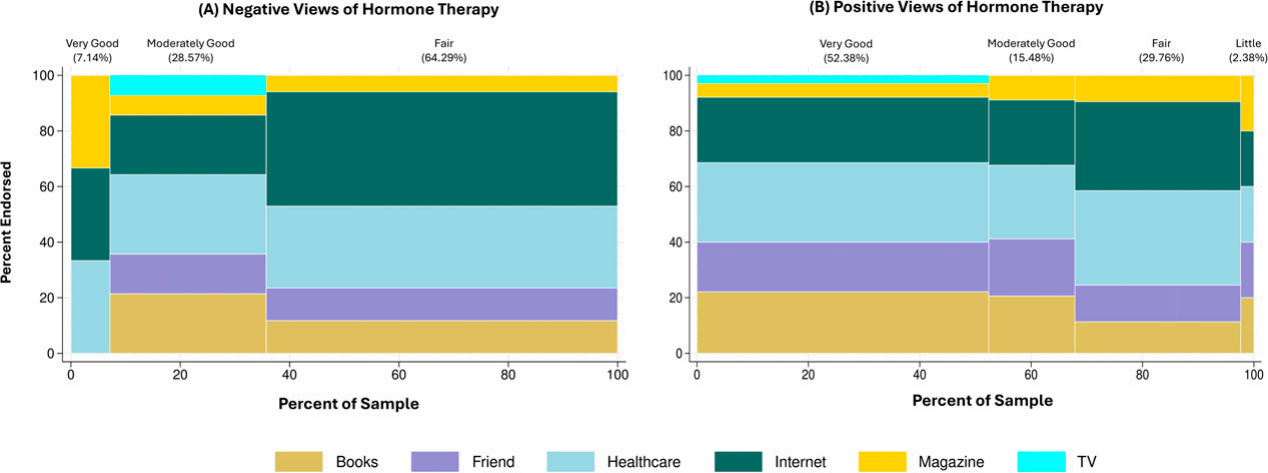
Information sources of menopausal knowledge level in individuals endorsing. **(A)** Negative views of hormone therapy and **(B)** positive views of hormone therapy.

As is shown in Figure 3, among women with negative perceptions regarding HT use, none had a knowledge level of “little.” For those with “fair” knowledge, the internet was the most common information source (41.18%). Among those with “moderately good” knowledge, healthcare providers were the most reported source (28.57%). For those with “very good” knowledge, the internet, magazines, and healthcare providers were equally reported (33.33% each). Among those with positive perceptions toward HT use and “little” knowledge, books, the internet, magazines, friends, and healthcare providers were equally reported (20% each). For those with “fair,” “moderately good,” and “very good” knowledge, healthcare providers were the most reported source (33.96%, 26.47%, and 28.57%, respectively).

As Hispanic participants reported lower use of HT and less positive perceptions of HT compared with non-Hispanic White participants, we conducted additional exploratory analyses to examine racial/ethnic differences in subjective menopausal knowledge levels, educational attainment, and information sources related to menopause. Regarding menopausal knowledge, there were significant differences between Hispanic and non-Hispanic participants in Firth logistic regressions. Compared to those with “very good” levels of menopausal knowledge, both participants with “fair” (OR = 4.75, 95% CI = [1.05–21.46] *p* < 0.05) and “moderately good” (OR = 7.66, 95% CI = [1.51–38.76] *p* < 0.05) levels of knowledge had higher odds of being Hispanic rather than being non-Hispanic, suggesting that comparatively lower knowledge levels of menopause may be more prevalent among Hispanic participants in this sample. However, there were no differences in educational attainment or sources of menopausal information across races/ethnicities.

## Discussion

The aim of this research was to investigate the relationship between perceptions of menopause and HT on HT use in perimenopausal and postmenopausal women. While prior studies have examined factors influencing views on menopause and HT separately, few have investigated how these views jointly relate to actual HT use. Our study contributes to this gap by empirically testing the associations between views on menopause, HT views, and HT use within the same analytic framework. However, our findings did not support a direct association between views on menopause and HT views, nor between either of these factors and HT use. Although views on menopause and HT were not directly associated with therapy use or with each other, our findings do reveal interesting associations between certain demographic and clinical characteristics and both HT views and use.

In this cohort, which was composed primarily of postmenopausal women (93%), the majority (85%) of respondents endorsed positive views of HT. In addition, 71.43% of women in our study were highly educated. Gan et al. reported that knowledge of HT and education were significantly associated in a cross-sectional study of menopausal women (*n* = 404) living in Kuala Lumpur, such that those with higher levels of education had better knowledge.^39^ The high level of education in our sample may thus have influenced our results. Additionally, nearly a third of our sample (32.65%) had ever used HT. HT use was associated with older age, greater alcohol consumption, and positive views regarding HT. These findings replicate those from Ghali et al. who found that the variables that predicted HT usage were white race, alcohol intake, and knowledge about HT.^40^

In our study, current alcohol consumption was associated with higher odds of ever using HT and having positive perceptions of HT. This finding aligns with previous studies that have identified alcohol consumption as one of the predictors of HT use.^41–43^ This association may be explained by age-related psychosocial changes, as women may exhibit increased alcohol consumption as they age, potentially due to factors such as retirement, the “empty-nest” period, illness, and widowhood.^42^ Peltier et al. found that women experienced changes in alcohol consumption during menopausal transition, with increased likelihood of transitioning from nonexcessive to excessive drinking during peri- and postmenopausal stages.^44^ However, in our sample, neither age nor menstrual status was significantly associated with current drinking status. Alcohol consumption may also be associated with both HT use and perceptions, as light to moderate drinking is often regarded by some individuals as part of a healthy lifestyle.^45^ Tivis and Tivis showed that moderate drinkers among postmenopausal women made conscious decisions to take care of their health, including healthier dietary and exercise habits.^46^ In this vein, moderate drinkers may view HT positively or use HT as part of a proactive approach to health. Furthermore, individuals experiencing more or severe menopausal symptoms may rely on both HT and alcohol as strategies to manage those symptoms. It has been suggested that low-risk alcohol consumption may be protective against various menopausal symptoms during the menopausal transition.^47^ However, this relationship should be interpreted with caution, given the cross-sectional nature of our data and the binary classification of current drinking status. Further research is warranted to explore the more nuanced associations between alcohol use, HT use, and perceptions.

In our cohort, Hispanic women showed 87% lower odds of using HT and 81% lower odds of viewing HT use positively compared to non-Hispanic White women. These results are consistent with previous studies, where Hispanics tended to be less likely to use HT, while non-Hispanic Whites reported the highest rates of HT use.^48–50^ There are few studies regarding perceptions of HT among Hispanics. However, Cortés and colleagues reported that 60.7% of their sample of Hispanic women viewed HT negatively.^51^ These ethnic discrepancies in HT use and perceptions may be due to several factors, including knowledge levels regarding menopause or HT. Previous studies have found that Hispanics report lower levels of HT knowledge,^50^ and Cortés et al. showed that 57.1% of Hispanic women reported having a little level of knowledge regarding menopause. In line with these findings, our cohort also indicates that comparatively lower knowledge levels about menopause may be more prevalent among Hispanic participants compared to non-Hispanic counterparts. While some researchers have put forth that these knowledge gaps may stem from educational disparities across race/ethnicity, as less educated women are generally less likely to report high HT knowledge. These knowledge gaps may stem from educational disparities across race/ethnicity, as less educated women are generally less likely to report high HT knowledge.^50^ In our sample, there were no differences in educational attainment across racial/ethnic groups. Thus, at least in our small sample, these differences do not appear to be attributable to educational differences, which is in line with another small study among low-income Hispanic women^52^ found that education level did not influence menopausal knowledge. Similarly, in our cohort, the sources of menopausal information are unlikely to be associated with discrepancies in menopausal knowledge, as the patterns of information sources related to menopause were fairly similar across racial/ethnic groups.

The question of why such ethnic/racial differences of menopausal knowledge, HT perceptions and use exist, thus, remains an open question that would benefit from future study with larger samples, and perhaps with qualitative or quantitative data that examine other factors beyond education level and sources of menopausal information. Cultural beliefs may play a role, with Hispanic women possibly preferring more “natural” alternatives and complementary treatments over HT. For example, Cortés et al. found that Hispanic participants opted for self-management alternatives and expressed reservations about HT.^51^ Christmas et al. also found that Hispanic women showed much higher use of herbal remedies for menopause management.^48^ Additionally, other factors may contribute to ethnic differences, such as insurance coverage,^49^ biological variations influencing physician prescribing patterns,^53^ and difference in symptom presentations.^50^

## Limitations

While these data provide evidence that there are ethnic differences in views of HT that may influence choices to use HT, there are several limitations. First, our data are not longitudinal, and thus we cannot make causal inferences about the direction of these effects. For example, it is possible that having a negative view of HT does not influence the decision of whether or not to use it, and more broadly that views of HT are unrelated to the decision to use it (as is evidenced by the fact that half of women who had positive views of HT were not users). Second, we did not specify the time period about views on menopause were queried, and instead just asked for general views.^27^ Thus, there may be recall bias. Finally, our sample was relatively small. Because of this, although we found that race/ethnicity moderated views of HT and menopause, it should be noted that our sample contained only 14 women who identified as Hispanic/Latina. Thus, caution should be used when interpreting these results and larger, ethnically diverse cohorts should be queried.

Despite these limitations, given the potential for HT to mitigate health problems that women are at a higher risk for in late life, including cardiovascular disease,^14,19,20^ understanding factors that influence decisions to initiate treatment is critically important, and understudied. Indeed, relatively few studies have examined views of menopause, HT, symptoms, sources of information, and HT use in conjunction. Furthermore, few studies examining these factors have been conducted recently (post-WHI data release) in America (although a number of studies exist abroad). Views about HT and menopause in America may be especially unique due to the nonhomogenous demographics. In our study, we found that Hispanic women were more likely than their White counterparts to hold negative views of HT, and that they also used HT to a lesser extent, highlighting a potential disparity in preventative health strategies that could have long-term consequences on health outcomes.

## Conclusion

A majority of peri- and postmenopausal women in the SAGE cohort demonstrated positive perceptions of both menopause and HT use. Older age, being Hispanic, being current drinkers, having lower level of menopausal knowledge and having vasomotor and genitourinary symptoms were significantly associated with HT use and positive HT perceptions. Hispanic participants showed lower levels of menopausal knowledge, less positive HT perceptions and lower HT use compared with non-Hispanic counterparts.

## Author Disclosure Statement

No competing financial interests exist.

## Abbreviations Used

AMH: Anti-Müllerian hormone
CEE: Conjugated equine estrogen
CI: Confidence interval
CIMT: Carotid artery intima-media thickness
E2: 17β estradiol
EPAT: Estrogen in Prevention of Atherosclerosis Trial
FSH: Follicle-stimulating hormone
GABA: γ-aminobutyric acid
HT: Hormone therapy
MRI: Magnetic Resonance Imaging
OR: Odds ratio
P4: Progesterone
SAGE: Sex, ApoE-4, γ-aminobutyric acid, and Episodic memory
SHBG: Sex hormone-binding globulin
STRAW: Stages of Reproductive Aging Workshop
SWAN: Study of Women's health Across the Nation
WHI: Women's Health Initiative
